# DYRK1A, a Novel Determinant of the Methionine-Homocysteine Cycle in Different Mouse Models Overexpressing this Down-Syndrome-Associated Kinase

**DOI:** 10.1371/journal.pone.0007540

**Published:** 2009-10-21

**Authors:** Christophe Noll, Chris Planque, Clémentine Ripoll, Fayçal Guedj, Anna Diez, Véronique Ducros, Nicole Belin, Arnaud Duchon, Jean-Louis Paul, Anne Badel, Bénédicte de Freminville, Yann Grattau, Henri Bléhaut, Yann Herault, Nathalie Janel, Jean-Maurice Delabar

**Affiliations:** 1 University Paris Diderot-CNRS EAC 4413, Unit of Functional and Adaptive Biology (BFA), Paris, France; 2 Département de Biologie Intégrée, unité fonctionnelle de nutrition, CHU Grenoble, Grenoble, France; 3 UMR6218 CNRS, Immunology and Molecular Embryology, UPS44, Institut de Transgenose, Orléans, France; 4 AP-HP, Hôpital Européen Georges Pompidou, Service de Biochimie, Paris, France; 5 Université Paris-Sud, UMR 1154-INRA, Faculté de Pharmacie, Châtenay-Malabry, France; 6 UMR-S 973, molécule thérapeutique in silico, University Paris Diderot, Paris, France; 7 CHU-Hôpital Nord – Service de Génétique, Saint Etienne, France; 8 Institut Jérôme Lejeune, Paris, France; 9 Fondation Jérome Lejeune, Paris, France; University of Western Ontario, Canada

## Abstract

**Background:**

Hyperhomocysteinemia, characterized by increased plasma homocysteine level, is associated with an increased risk of atherosclerosis. On the contrary, patients with Down syndrome appear to be protected from the development of atherosclerosis. We previously found a deleterious effect of hyperhomocysteinemia on expression of DYRK1A, a Down-syndrome-associated kinase. As increased expression of DYRK1A and low plasma homocysteine level have been associated with Down syndrome, we aimed to analyze the effect of its over-expression on homocysteine metabolism in mice.

**Methodology/Principal Findings:**

Effects of DYRK1A over-expression were examined by biochemical analysis of methionine metabolites, real-time quantitative reverse-transcription polymerase chain reaction, and enzyme activities. We found that over-expression of Dyrk1a increased the hepatic NAD(P)H:quinone oxidoreductase and S-adenosylhomocysteine hydrolase activities, concomitant with decreased level of plasma homocysteine in three mice models overexpressing Dyrk1a. Moreover, these effects were abolished by treatment with harmine, the most potent and specific inhibitor of Dyrk1a. The increased NAD(P)H:quinone oxidoreductase and S-adenosylhomocysteine hydrolase activities were also found in lymphoblastoid cell lines from patients with Down syndrome.

**Conclusions/Significance:**

Our results might give clues to understand the protective effect of Down syndrome against vascular defect through a decrease of homocysteine level by DYRK1A over-expression. They reveal a link between the Dyrk1a signaling pathway and the homocysteine cycle.

## Introduction

Homocysteine (Hcy) is a sulfur-containing amino acid formed during the intracellular conversion of methionine via the adenosylated compounds S-adenosylmethionine (SAM) and S-adenosylhomocysteine (SAH). The formation of SAM is catabolized by methionine adenosyl transferase (MAT). Once Hcy is formed, it may be recycled to methionine after remethylation by two different pathways. The first one involves methionine synthase (MS), an enzyme that uses vitamin B_12_ (cobalamin) as an essential cofactor and 5-methyltetrahydrofolate as the methyl donor. The 5-methyltetrahydrofolate is generated by 5, 10-methylene tetrahydrofolate reductase (MTHFR) [1]. The second pathway, which occurs in liver and kidney, involves the enzyme betaine-homocysteine methyltransferase (BHMT). Hcy may also undergo condensation with serine to form cystathionine, which is catalyzed by the vitamin B_6_-dependent enzyme cystathionine beta synthase (CBS), the first enzyme involved in the transsulfuration pathway. Cystathionine is subsequently hydrolysed to form cysteine which can be, in turn, incorporated into protein or used to synthesize the antioxidant glutathione. Hcy can also turn back to SAH via reversal of the SAH hydrolase (SAHH) reaction [1].

Elevated plasma Hcy levels are well-recognized as an independant risk factor for atherosclerosis in the coronary, cerebrovascular and peripheral arterial circulation [2]. Conversely, although Down syndrome (DS) is associated with a great variety of phenotypes, the incidence of atherosclerotic vascular disease seems to be low [3], [4]. Even if the coronary arteries of DS patients were not completely free of atherosclerosis, it was milder than in other mentally retarded patients and in control subjects of the same age [5]. Moreover, healthy old DS patients showed some classical biochemical risk factors for atherosclerosis but did not suffer from clinical cardiovascular alterations [6]. Because many genetic factors can be related to this reduction, the reasons for this apparent protection against atherosclerosis remain unclear. DYRK1A, which gene is localized on human chromosome 21, is a protein kinase that belongs to an evolutionarily conserved family of proteins known as DYRKs (dual-specificity tyrosine-(Y)-phosphorylation regulated kinase) involved in diverse functions ranging from development, growth to apoptosis [7]–[9]. On the one hand, we recently reported a reduction of Dyrk1a protein level in liver of CBS-deficient mice, a murine model of hyperhomocysteinemia [10], suggesting a link between DYRK1A related pathways and the Hcy cycle. On the other hand, an increased expression of DYRK1A and low plasma Hcy levels have been associated with DS [6], [11]–[13]. To analyze further the relation between DYRK1A and Hcy metabolism, we used four transgenic models to demonstrate the effect of the over-expression of Dyrk1a on Hcy metabolism: a model of hyperhomocysteinemia due to CBS deficiency [14] and three models with duplications of increasing complexity and over-expression of Dyrk1a [15]: a BAC transgenic with one copy of the murine Dyrk1a gene; a YAC transgenic for a human chromosome 21 fragment carrying five genes including DYRK1A; a partial trisomy 16 mouse carrying an extra copy of a region of MMU16 syntenic for a region of HSA21 between Mrpl39 and Znf295 containing 138 genes (also including Dyrk1a) and considered to be a valid mouse model of human Down syndrome [16].

## Results

### DYRK1A over-expression reduces the plasma Hcy levels in mice

**Figure 1:**
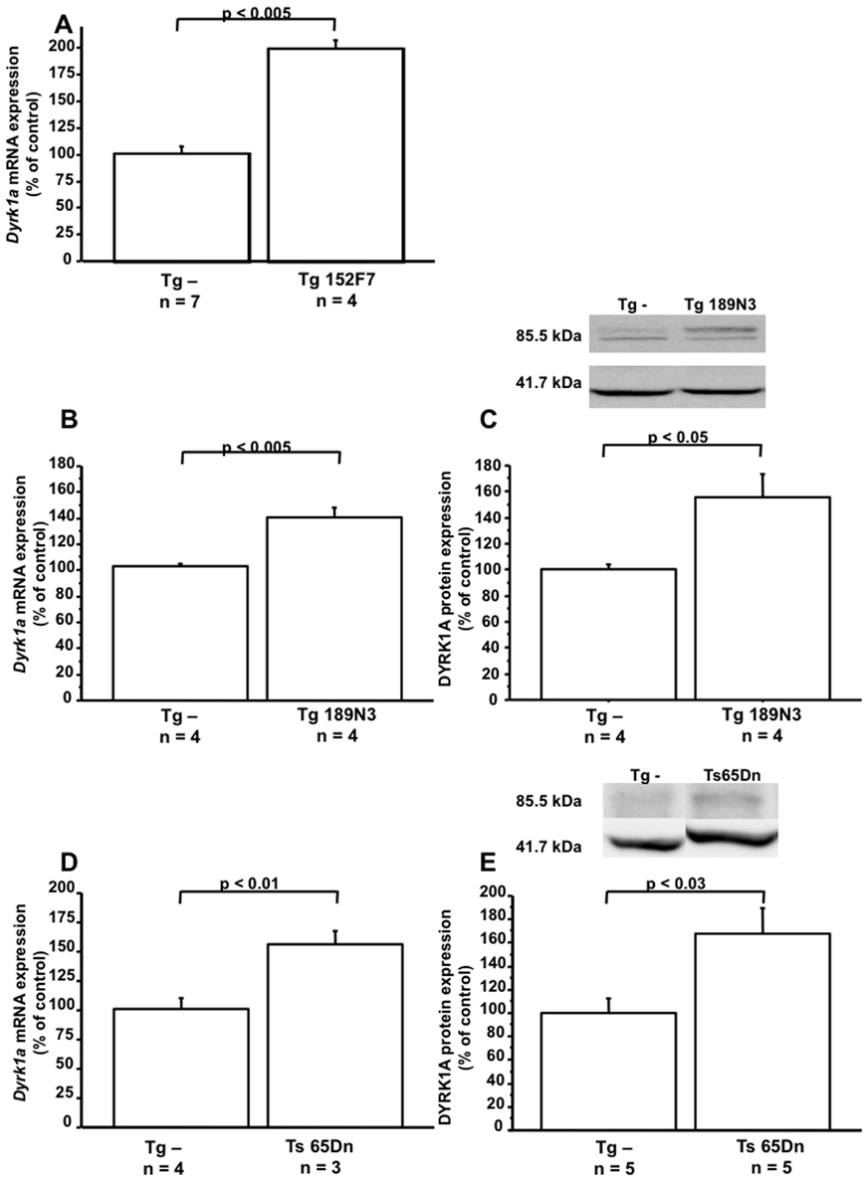
Hepatic DYRK1A mRNA and protein expression in liver of transgenic mice. Relative expression of DYRK1A gene was based on Q-PCR data and protein expression was determined by normalization of the density of images from DYRK1A with that of β-actin of the same blot. The values of Tg 152F7, Tg189N3 and Ts65Dn were normalized to the mean Tg – mice from each lines. The blots are representative of three independent experiments. Data correspond to means ± SEM and the statistical analysis was done by Student's unpaired *t*-tests. n = number of mice.

**Figure 2:**
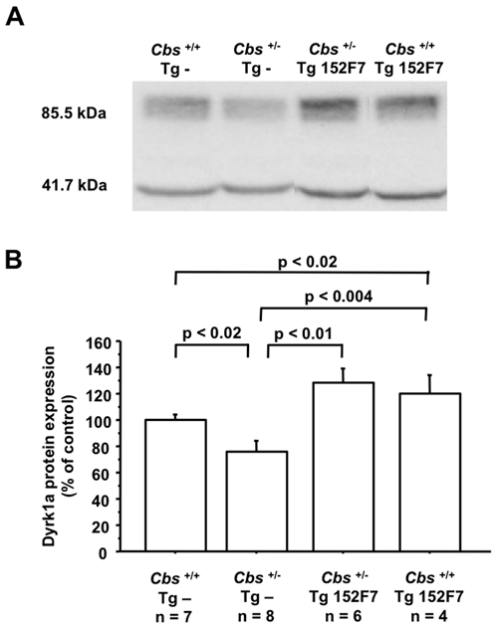
DYRK1A protein expression in liver of CBS-deficient mice crossbred with 152F7 transgenic mice. (A) Western immunoblots showing DYRK1A expression in liver of wild type mice (*Cbs* ^+/+^ Tg -), heterozygous mice (*Cbs* ^+/−^ Tg -), 152F7 transgenic mice (*Cbs* ^+/+^ Tg 152F7), and heterozygous mice crossbred with 152F7 transgenic mice (*Cbs* ^+/−^ Tg 152F7). Proteins were subjected to immunoblot analysis using antibodies specific to DYRK1A (85.5 kDa). After stripping, the membranes were reprobed with anti-β-actin antibody (41.7 kDa) for the control. (B) Relative protein expression was determined by normalization of the density of images from DYRK1A with that of β-actin of the same blot. The values of *Cbs* ^+/−^ Tg -, *Cbs* ^+/+^ Tg 152F7, or *Cbs* ^+/−^ Tg 152F7 were normalized to the mean of *Cbs* ^+/+^ Tg - mice. The blots are representative of three independent experiments. Data correspond to means ± SEM and the statistical analysis was done with one-way ANOVA followed by Student's unpaired *t*-tests. n = number of mice.

In order to analyze the over-expression of DYRK1A on plasma Hcy levels, we used three models of mice, which overexpress not only the murine gene, but also the human one. The transgenic line (Tg) 152F7 contains five human genes including DYRK1A. The Tg 189N3 contains the murine orthologue of DYRK1A. The Ts65Dn line is the most complete of the commonly available mouse models of the mouse partial trisomy 16 and exhibits morphological and biochemical changes seen in DS [17], [18]. We first analyzed the overexpression of DYRK1A in liver of mice. Male Tg 152F7 mice, two months of age, showed a two-fold increase in gene expression of DYRK1A in the liver compared to non-transgenic mice (Fig. 1A). Female Tg 189N3 mice, two months of age, showed a 1.4-fold increase in gene expression of *Dyrk1a* compared to non-transgenic mice (Fig. 1B). Male Ts65Dn mice, six months of age, showed a 1.6-fold increase in gene expression of *Dyrk1a* compared to non-transgenic mice (Fig. 1D). Commensurate with the mRNA expression, protein expression of DYRK1A was 1.3 fold, 1.6 fold and 1.7 fold higher in liver of Tg 152F7, Tg 189N3, and Ts65Dn mice respectively (Fig. 2 and Figs. 1C, 1E).

**Figure 3:**
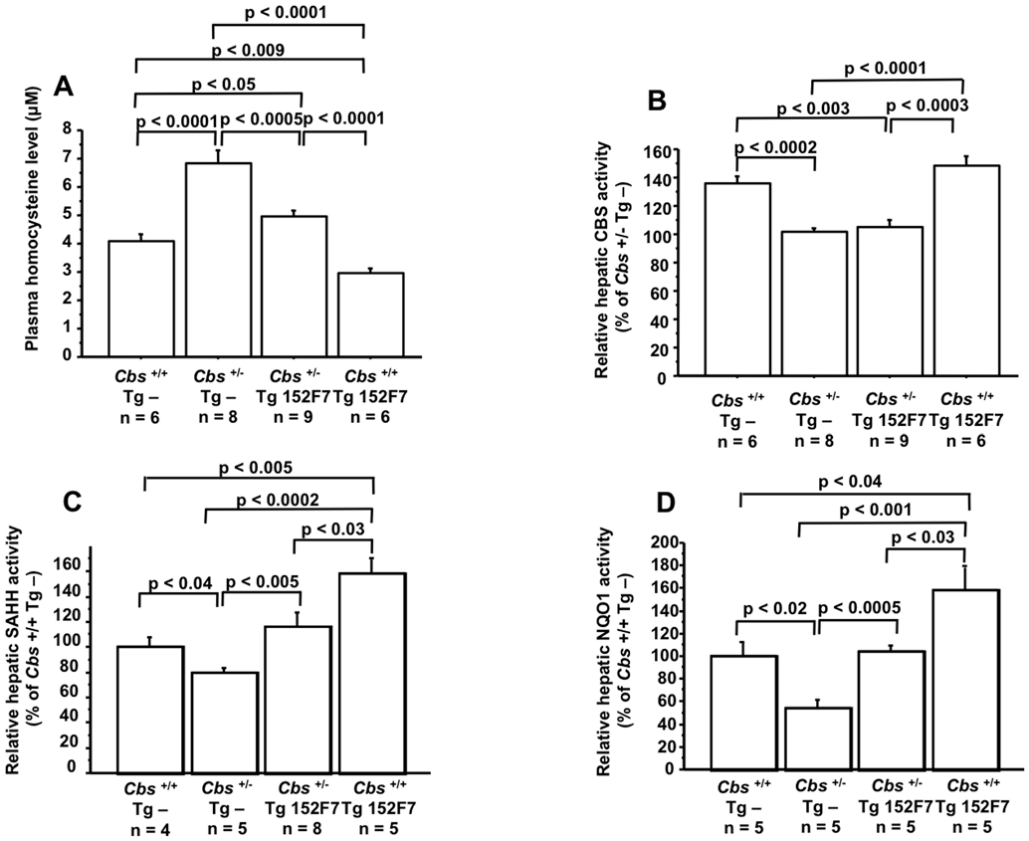
Plasma Hcy level is decreased in 152F7 transgenic mice and in CBS-deficient mice crossbred with 152F7 transgenic mice. (A) Plasma Hcy level, (B) hepatic CBS, (C) SAHH and (D) NQO1 activity in wild type mice (*Cbs* ^+/+^ Tg -), heterozygous mice (*Cbs* ^+/−^ Tg -), 152F7 transgenic mice (*Cbs* ^+/+^ Tg 152F7), and heterozygous mice crossbred with 152F7 transgenic mice (*Cbs* ^+/−^ Tg 152F7). The values were normalized to the mean of *Cbs* ^+/+^ Tg - mice. Data correspond to means ± SEM and the statistical analysis was done with one-way ANOVA followed by Student's unpaired *t*-tests. n = number of mice.

In order to show the effect of over-expression of DYRK1A on plasma Hcy level, serum of Tg 152F7 and heterozygous CBS-deficient (*Cbs* ^+/−^) mice crossbred with Tg 152F7 mice was analyzed by HPLC. Tg 152F7 mice (*Cbs* ^+/+^ Tg 152F7; Fig. 3A and Table 1) have a Hcy level 1.4-fold lower than those of *Cbs* ^+/+^ mice (*Cbs* ^+/+^ Tg -; Fig. 3A and Table 1). As previously shown [10], Dyrk1a protein expression was decreased in liver of *Cbs* ^+/−^ mice (*Cbs* ^+/−^ Tg -; Fig. 2), compared with protein extracted from *Cbs* ^+/+^ mice (*Cbs* ^+/+^ Tg -; Fig. 2). *Cbs* ^+/−^ mice crossbred with Tg 152F7 mice (*Cbs* ^+/−^ Tg 152F7; Fig. 2) also showed a 1.3 fold-increased hepatic expression of DYRK1A compared to *Cbs* ^+/−^ mice (*Cbs* ^+/−^ Tg -; Fig. 2). As expected, Hcy level of *Cbs* ^+/−^ mice (*Cbs* ^+/−^ Tg -; Fig. 3A) was 1.7-fold higher than those of *Cbs* ^+/+^ mice (*Cbs* ^+/+^ Tg -; Fig. 3A). However, *Cbs* ^+/−^ mice crossbred with Tg 152F7 mice (*Cbs* ^+/−^ Tg 152F7; Fig. 3A) have a Hcy level 1.4-fold lower than those of *Cbs* ^+/−^ mice (*Cbs* ^+/−^ Tg -; Fig. 3A). Tg 189N3 and Ts65Dn mice have also a Hcy level 2.35-fold and 1.4-fold lower than those of non-transgenic mice respectively (Table 1). These results emphasize the effect of over-expression of DYRK1A on plasma Hcy level, not only in case of hyperhomocysteinemia but also in the context of DS.

**Table 1:** Plasma Hcy levels, hepatic SAM and SAH concentrations, and hepatic SAM/SAH ratio in transgenic mice.

Genotype (n = number of mice)	Hcy (µM)	SAM (nmol/g)	SAH (nmol/g)	SAM/SAH
Tg – (n = 4)	3.9±0.3	79.5±9.1	52.7±3.1	1.5±0.2
Tg 152F7 (n = 6)	2.9±0.2*	73.2±6.4	42.4±4.1	1.8±0.2
Tg – (n = 5)	4.7±0.6	64.8±10.3	35.7±3.3	1.8±0.2
Tg 189N3 (n = 5)	2±0.1**^‡^**	67.7 ±6.5	35.5±3.1	2±0.2
Tg – (n = 7)	2.6±0.1	52±3.8	44±3.1	1.2±0.1
Ts65Dn (n = 6)	1.9±0.3*	35.6±3.5**^†^**	51.4±3.8	0.7±0.1**^†^**

Data correspond to means ± SEM and the statistical analysis was done by Student's unpaired *t*-tests. * *p*<0.04; **^†^** *p*<0.01; **^‡^** *p*<0.002.

### DYRK1A over-expression modulates SAHH activity

As CBS is a key enzyme of Hcy metabolism [19], [20], we first assayed the CBS activity in liver samples of mice. As expected, *Cbs* ^+/−^ mice (*Cbs* ^+/−^ Tg -; Fig. 3B) showed a significant decrease of CBS activity when compared with *Cbs* ^+/+^ mice (*Cbs* ^+/+^ Tg -; Fig. 3B). Tg 152F7 mice (*Cbs* ^+/+^ Tg 152F7; Fig. 3B) have the same CBS activity than that of *Cbs* ^+/+^ mice (*Cbs* ^+/+^ Tg -; Fig. 3B). However, *Cbs* ^+/−^ mice crossbred with Tg 152F7 mice (*Cbs* ^+/−^ Tg 152F7; Fig. 3B) showed a decrease of CBS activity like *Cbs* ^+/−^ mice when compared with *Cbs* ^+/+^ mice. Tg 189N3 (110±4 *versus* 100±7; *p*<0.26 by Student's *t* test *n* = 5 for each) and Ts65Dn (111±7 *versus* 100±3; *p*<0.17 by Student's *t* test *n* = 5 for each) mice have also the same CBS activity than that of non-transgenic mice. Then our results show that dyrk1a overexpression has no effect on CBS activity.

**Figure 4:**
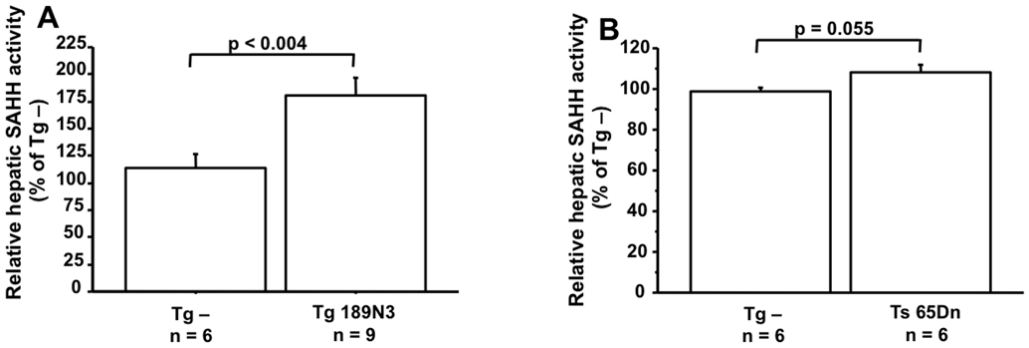
Hepatic SAHH activity is increased in Tg 189N3 and Ts65Dn transgenic mice. SAHH activity in (A) Tg 189N3 and (B) Ts65Dn transgenic mice. The values were normalized to the mean of Tg – mice from each line. Data correspond to means ± SEM and the statistical analysis was done by Student's unpaired *t*-test. n = number of mice.

As Hcy can revert back to SAH via the SAHH mediated reverse reaction, we assayed SAHH activity in mouse liver. Tg 152F7 mice (*Cbs* ^+/+^ Tg 152F7; Fig. 3C) showed an increase of SAHH activity when compared with *Cbs* ^+/+^ mice (*Cbs* ^+/+^ Tg -; Fig. 3C). Moreover, *Cbs* ^+/−^ mice crossbred with Tg 152F7 mice (*Cbs* ^+/−^ Tg 152F7; Fig. 3C) counteracted the decreased SAHH activity when compared with *Cbs* ^+/−^ mice (*Cbs* ^+/−^ Tg -; Fig. 3C). Tg 189N3 (Fig. 4A) and Ts65Dn (Fig. 4B) mice also showed an increased SAHH activity when compared to non-transgenic mice. We also determined the hepatic levels of SAM and SAH in Tg mice overexpressing DYRK1A and found that levels of SAM and SAH in liver of Tg 152F7 and Tg189N3 did not differ from non-transgenic mice (Table 1). However, we found a decreased level of SAM in liver of Ts65Dn mice compared to non-transgenic mice, leading to decreased SAM to SAH ratio (Table 1).

In order to determine if other enzymes of the methionine and folate cycles are altered, we assayed the mRNA expression of the major enzymes involved in the metabolism of Hcy in the liver of Tg 189N3. We found that even if the *Mat1a* mRNA expression was not affected in Tg 189N3 mice (data not shown), the *Bhmt*, *Ms* and *Mthfr* mRNA expression were increased compared to non-transgenic mice (Table 2). However, the *Sahh* mRNA expression was decreased in Tg 189N3 mice compared to non-transgenic mice (Table 2).

**Table 2:** Relative expression of SAHH, BHMT, MS and MTHFR gene based upon Q-PCR data obtained from non-transgenic and Tg189N3 mice.

Genotype (n = number of mice)	SAHH (% of control)	BHMT (% of control)	MS (% of control)	MTHFR (% of control)
Tg – (n = 4)	104±14	100±6	100±4	103±11
Tg 189N3 (n = 4)	55±12**^†^**	242±45**^†^**	891±195**^†^**	726±330*

The values of Tg189N3 were normalized to the mean Tg – mice. Data correspond to means ± SEM and the statistical analysis was done by Student's unpaired *t*-tests. n = number of mice. *p = 0.068; **^†^** *p*< 0.03.

### Plasma Hcy level is negatively correlated with hepatic DYRK1A expression and SAHH activity

**Figure 5:**
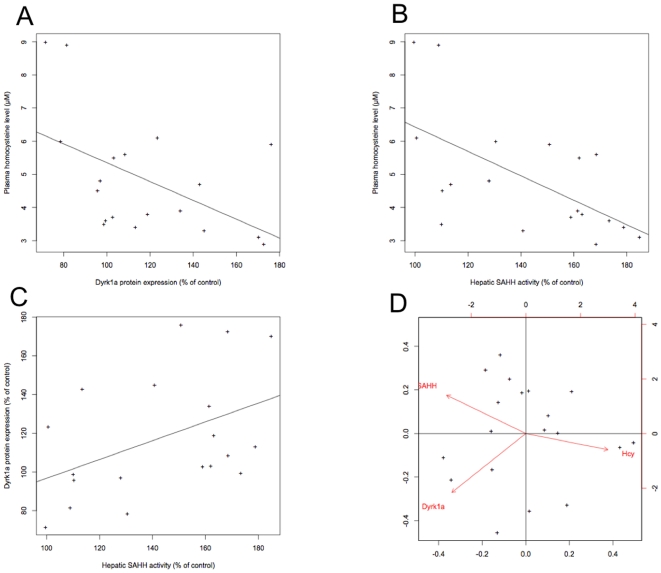
Plasma Hcy level, DYRK1A protein expression and SAHH activity are correlated. Hepatic DYRK1A expression and SAHH activity are presented as percent of *Cbs* ^+/+^ Tg – mice. Correlation of plasma Hcy level vs. (A) hepatic DYRK1A protein expression or (B) hepatic SAHH activity. Increasing levels of plasma Hcy and hepatic DYRK1A protein expression or SAHH activity are negatively correlated at *p*<0.04 and *p*<0.006 with a ρ = −0.48 and ρ = −0.61 respectively. Correlation of hepatic DYRK1A protein expression vs. SAHH activity (C). Increasing levels of hepatic DYRK1A protein expression and hepatic SAHH activity are positively correlated at *p*<0.05 with a ρ = 0.47. (D) Graph of PCA. The three quantitative variables corresponding of plasma Hcy level, hepatic DYRK1A protein expression and hepatic SAHH activity are represented by vectors.

We previously observed a negative correlation between plasma Hcy levels and hepatic DYRK1A expression, which emphasizes the effect of hyperhomocysteinemia on DYRK1A expression [10]. Then we investigated if there are any links between Hcy concentration, hepatic DYRK1A protein expression and hepatic SAHH activity. We observed a significant negative correlation between plasma Hcy level and not only hepatic DYRK1A protein expression (ρ = −0.48, p<0.04; Fig. 5A) but also hepatic SAHH activity (ρ = −0.61, p<0.006; Fig. 5B). Moreover, hepatic DYRK1A protein expression was also positively correlated with hepatic SAHH activity (ρ = 0.47, p<0.05; Fig. 5C). Multivariate analysis revealed a negative correlation between plasma Hcy level and hepatic DYRK1A protein expression and SAHH activity (Fig. 5D), indicating that high Hcy levels are associated with low hepatic DYRK1A protein expression and low hepatic SAHH activity in mice.

### DYRK1A over-expression modulates NQO1 activity

We found that the *Sahh* mRNA expression not only was decreased in Tg 189N3 mice (Table 2) but also in Ts65Dn (69.6±5.6 *versus* 102.1±8.5; *p*<0.005 by Student's *t* test *n* = 4 for each mouse) compared to non-transgenic mice. The increased activity of NAD(P)H:quinone oxidoreductase (NQO1) is followed by an increase of a by-product of the enzyme reaction, NAD+, which is a cofactor of SAHH [21]. In order to determine if SAHH is a direct or an indirect target of Dyrk1a, we analyzed the activity of NQO1 in liver of transgenic mice. We found an increased activity of NQO1 not only in Tg152F7 (*Cbs* ^+/+^ Tg 152F7; Fig. 3D), Tg 189N3 but also in Ts65Dn mice compared to non-transgenic mice (Fig. 3D and Table 3). Moreover, *Cbs* ^+/−^ mice crossbred with Tg 152F7 mice (*Cbs* ^+/−^ Tg 152F7; Fig. 3D) counteracted the decreased NQO1 activity when compared with *Cbs* ^+/−^ mice (*Cbs* ^+/−^ Tg -; Fig. 3D). Commensurate with the increased activity, mRNA expression of NQO1 was 1.8 fold higher in liver of Tg 189N3 and Ts65Dn mice compared to non-transgenic mice (Table 3).

**Table 3:** Relative expression of NQO1 gene based upon Q-PCR data and NQO1 activity obtained from non-transgenic and Tg189N3 and Ts65Dn mice.

Genotype (n = number of mice)	mRNA expression (% of control)	activity (% of control)
Tg -	100.7±4 (n = 4)	100±20,9 (n = 3)
Tg 189N3	177.3±37.3* (n = 3)	379.5 ±74.2* (n = 4)
Tg –	100.9±5 (n = 4)	100±27,2 (n = 5)
Ts65Dn	176.3±22.6**^†^** (n = 4)	212±32,5* (n = 5)

The values of Tg189N3 and Ts65Dn were normalized to the mean Tg – mice from each line. Data correspond to means ± SEM and the statistical analysis was done by Student's unpaired *t*-tests. n = number of mice. *p<0.03; **^†^** *p*<0.01.

### Harmine inhibits the effects of DYRK1A over-expression on SAHH activity

**Figure 6:**
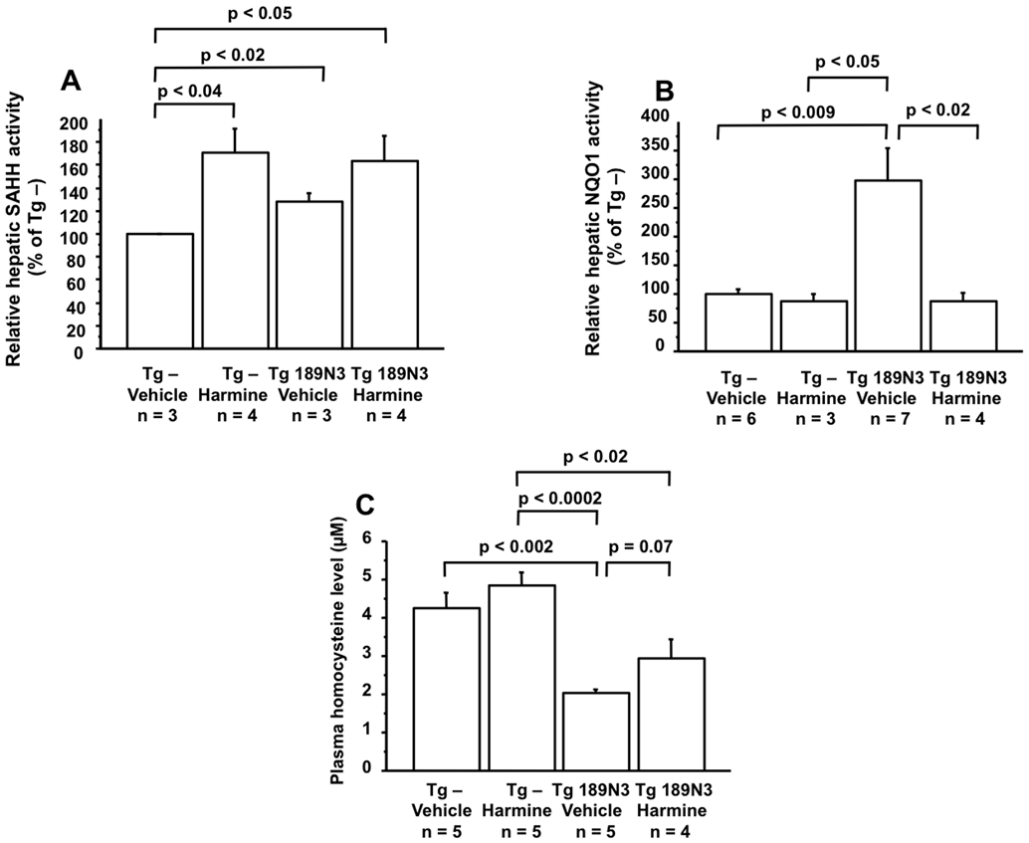
Effects of harmine on hepatic SAHH and NQO1 activities, and on plasma Hcy levels in Tg 189N3 mice. Hepatic SAHH (A) and NQO1 (B) activities are presented as percent of untreated (Vehicle) non-transgenic (Tg –) mice activities. (C) Plasma Hcy level. Data correspond to means ± SEM and the statistical analysis was done with one-way ANOVA followed by Student's unpaired *t*-test. n = number of mice.

In order to determine if the effects are dependent on Dyrk1a kinase activity, we treated Tg 189N3 mice with harmine, the most potent and specific inhibitor of Dyrk1a [22], [23]. Even non-transgenic mice treated with harmine (Tg – Harmine, Fig. 6A) showed an increased SAHH activity compared to untreated non-transgenic mice (Tg – Vehicle, Fig. 6A), Tg 189N3 mice treated with harmine (Tg 189N3 Harmine, Fig. 6A) had the same increased activity than that of non-transgenic mice treated with harmine (Tg – Harmine, Fig. 6A), which shows that treatment with harmine prevents the increase of SAHH activity in Tg 189N3 mice. Moreover, even if harmine treatment has no effect on NQO1 in non transgenic mice (Tg – Harmine, Fig. 6B), treatment with harmine in Tg 189N3 mice (Tg 189N3 Harmine, Fig. 6B) reduced NQO1 activity compared to non treated Tg 189N3 mice (Tg 189N3 Vehicle, Fig. 6B).

To confirm the implication of Dyrk1a on Hcy level, we also assayed plasma Hcy levels in harmine-treated non-transgenic and Tg 189N3 mice. There was a trend towards a 1.4 fold increase of plasma Hcy concentration in harmine treated Tg 189N3 (Tg 189N3 Harmine, Fig. 6C) compared to non-treated Tg 189N3 mice (Tg 189N3 Vehicle, Fig. 6C) although this was not statistically significant.

### SAHH and NQO1 activities were increased in lymphoblastoid cell lines

In order to determine whether the activities observed in mice does apply to humans, we also analyzed the SAHH and NQO1 activity in lymphoblastoid cell lines (LCLs) from patients with DS. We first confirmed that LCLs from patients with DS (T21, Table 4) overexpressed 1.5 fold DYRK1A at the mRNA and protein level compared to LCLs from control individuals (control, Table 4). We not only found an increased SAHH activity but also a decreased mRNA *SAHH* expression in LCLs from patients with DS compared to LCLs from control individuals (Table 4). Moreover, the NQO1 activity was also increased in LCLs from patients with DS, concomitant with an increased mRNA expression (Table 4).

**Table 4:** Relative expression of DYRK1A, SAHH and NQO1 and SAHH and NQO1 activities obtained from lymphoblastoid cell lines.

Genotype	DYRK1A mRNA expression (% of control)	DYRK1A protein expression (% of control)	SAHH mRNA expression (% of control)	SAHH activity (% of control)	NQO1 mRNA expression (% of control)	NQO1 activity (% of control)
control	100±9.3 (n = 12)	100±9.6 (n = 16)	100±19.3 (n = 12)	100±8.7 (n = 10)	99.9±11 (n = 11)	100±16 (n = 6)
T21	150.8±10.3**^†^** (n = 12)	154.3±15.6**^†^** (n = 16)	53.8±6.5* (n = 12)	135.5±12.8* (n = 12)	160.9±14.6**^†^** (n = 11)	228±55* (n = 6)

The values of lymphoblastoid cell lines (LCLs) from patients with DS (T21) were normalized to the mean lymphoblastoid cell lines from control individuals (control). Data correspond to means ± SEM and the statistical analysis was done by Student's unpaired *t*-tests. n = number of LCLs. * *p*<0.05; **^†^** *p*<0.006.

## Discussion

Elevated plasma Hcy level is well recognized as an important vascular risk factor and atherosclerosis in the coronary, cerebrovascular and peripheral arterial circulation, even if the degree of hyperhomocysteinemia is moderate [2]. On the contrary, adults with Down syndrome seem to be protected against atherosclerosis, despite having elevated risk factors, such as elevated total body fat, higher levels of triglycerides and C-reactive protein, and lower levels of moderate to vigorous physical activity [24]. As we found a negative correlation between plasma Hcy levels and hepatic DYRK1A expression which underlines the effect of hyperhomocysteinemia on DYRK1A expression [10], we decided to analyze the over-expression of DYRK1A on Hcy metabolism. Here we show that overexpression of DYRK1A diminishes the plasma Hcy level. This result was obtained with different murine models which contain the human or the murine gene, with duplications of increasing complexity and over-expression of Dyrk1a. Therefore, we see an effect of Hcy level on DYRK1A expression [10], but also an effect of DYRK1A expression on Hcy level, which is underlined by the correlation between plasma Hcy level and hepatic DYRK1A protein expression (10 and Figure 5).

As increased expression of DYRK1A and low plasma Hcy levels have been associated with DS [6], [11]–[13], we also analyzed the contribution of DYRK1A on Hcy levels in a mouse trisomic complex, the Ts65Dn mice. We found an increased expression of DYRK1A in liver and a decreased level of plasma Hcy level in Ts65Dn. *DYRK1A* and *CBS* are two genes located on chromosome 21. Our results show that over-expression of DYRK1A has no effect on CBS activity, which is in agreement with the hepatic SAM levels (an allosteric activator of CBS). It has been demonstrated that DS children have increased cystathionine level relative to normal children, consistent with over-expression of the CBS gene present on human chromosome 21 and mouse chromosome 17 [11]. On the one hand, the over-expression of CBS in DS is the predominant mechanism to explain the decrased plasma Hcy level in DS patients [11]. On the other hand, the DYRK1A over-expression likely plays a role on decreased plasma Hcy level in DS patients however, to a lesser extent.

We showed that over-expression of DYRK1A increases the SAHH activity not only in mice, but also in LCLs obtained from DS patients. Moreover, treatment with harmine, the most potent and specific inhibitor of Dyrk1a [22], [23], demonstrates that the increased SAHH activity depends on Dyrk1A kinase activity. We also established a correlation between plasma Hcy level, hepatic DYRK1A protein expression and hepatic SAHH activity. However, the increased hepatic SAHH activity was not associated with statistical difference in hepatic SAH levels. A previous study showed a lower concentration of plasma Hcy and SAH levels in DS children than in plasma of control subjects [11]. Galletti et al. also demonstrated that the intracellular concentration of SAH is significantly reduced in DS erythrocytes, paralleling the low plasma Hcy levels, and the intracellular SAM concentration is the same as in the control cells [13]. Therefore the diminution of SAH levels could be due to other genes located on chromosome 21. We found a decreased hepatic SAM levels, associated with a decrease of the SAM/SAH ratio in Ts65Dn, which suggests an altered SAM-dependent methylation, but not in Tg 152F7 and Tg 189N3 mice. Interestingly, Ts65Dn mice also contain three copies of DNMTa1 gene, which encodes for a DNA methyltransferase. The over-expression of this DNA methyltransferase could decrease SAM level and the SAM/SAH ratio in liver of Ts65Dn mice, which could influence the methylation index and then the gene expression.

SAHH cleaves SAH to adenosine and Hcy, but this reaction is easily reversible by the same enzyme [25]. Although the equilibrium dynamics of the SAHH reaction strongly favor SAH formation, under physiological conditions, the rapid metabolism of SAHH end products Hcy (via transsulfuration and remethylation pathways) and adenosine (via the adenosine deaminase or adenosine kinase) drive the SAHH reaction in the hydrolysis direction. The activation of the SAHH activity in the reverse direction would be expected to promote the diminution of Hcy. The finding that DYRK1A over-expression activated SAHH activity is novel and is also consistent with no limitation of the remethylation pathway. We found an increased expression of the three enzymes implicated in the remethylation pathway in liver of Tg189N3 mice. However, the SAHH expression was decreased not only in liver of Tg 189N3 and Ts65Dn mice, but also in LCLs from DS patients. Because SAHH transcripts were decreased, the finding of increased SAHH activity must represent a post-transcriptional modification of the enzyme. The activation of SAHH activity can be due to a direct or an indirect mechanism. We found an increased activity of NQO1 not only in liver of Tg 152F7, Tg 189N3 and Ts65Dn mice, but also in LCLs obtained from DS patients. Moreover, treatment with harmine demonstrates that the increased NQO1 activity depends on Dyrk1A kinase activity. The increased activity of NQO1 is followed by an increase of a by-product of the enzyme reaction, NAD+, which is a cofactor of SAHH [21]. NQO1 is an inducible enzyme, and its overexpression protects cells against cell death [26]. The cell's major strategy of coping with oxidative stress is to increase the anti-oxidative potential by upregulating defense enzymes through activation of the nuclear factor-E2-related factor-2 (Nrf2). Nrf2 can activate many phase II detoxifying and antioxidant genes including NQO1 [27]. Nrf2 dissociates from the cytoskeletal binding protein KEAP1 and translocates to the nucleus in response to oxidative stress. It has been demonstrated that the PI3K/Akt pathways facilitate the release of NRF2 from KEAP1 and its subsequent translocation, and regulate ROS-dependent Nrf2 activation [28]. Previous results have shown that phosphorylation of protein kinase Akt was increased in Ts65Dn mice, and we found an increased hepatic NQO1 activity in these mice [29]. Then over-expression of DYRK1A could modulate the hepatic SAHH activity through an indirect mechanism initiated by its serine/threonine kinase activity.

In conclusion, our results might give clues to understand the protective effect of DS against vascular defect through a decrease of Hcy level, and a link between metabolomics and signalling pathways.

## Methods

### Mice and genotyping

Mice were maintained in a controlled environment with unlimited access to food and water on 12 h light/dark cycle. All procedures were carried out in accordance with internal guidelines of the French Agriculture Ministry for animal handing. Number of mice and suffering were minimized as possible. Mice heterozygous for targeted disruption of the *Cbs* gene (*Cbs* ^+/−^) were generously donated by Dr. N. Maeda (Department of Pathology, University of North Carolina, Chaped Hill, NC, USA) [14]. *Cbs* ^+/−^ mice, on a C57BL/6 background were obtained by mating male *Cbs* ^+/−^ mice with female wild-type C57BL/6 (*Cbs* ^+/+^) mice. DNA isolated from 4 week-aged mice tail biopsies was subjected to genotyping of the targeted CBS allele using a polymerase chain reaction (PCR) assay [14]. The human yeast artificial chromosome 152 F7 (YAC-152F7) strain has been previously described [30]. This transgenic line (Tg 152F7) contains the genes PIGP, TTC3, DSCR9, DSCR3 and DYRK1A. The YAC-152F7 line, which was constructed in an FVB/N background, was backcrossed on a C57BL/6 background. Genotyping was performed by PCR using specific human primers [30]. *Cbs* ^+/−^ and Tg 152F7 mice, on the same background, were crossbred. Male from each genotype from the same litter, two months of age, were used. The murine bacterial artificial chromosome 189 N3 (BAC-189N3) strain has been constructed by electroporating HM-1 embryonic stem (ES) cells with the retrofitted BAC-189N3. ES clone was selected for overexpression of Dyrk1a, and injected into blastocysts (J Delabar, personal communication). Female Tg 189N3 mice and control from the same litter, two months of age, were used. Ts65Dn mice possess a third copy of a region of mouse chromosome 16 from *App* to *Mx1*, orthologous to the DS critical region of HSA 21 [31]. Male Ts65Dn mice and control, from the same litter, six months of age, were used.

### Harmine treatment

Mice were injected intraperitoneally overnight, with 10 mg/kg of harmine hydrochloride hydrate (Fisher Scientific, Illkirch, France) dissolved in 0.9% NaCl. The next morning, mice were injected once more for 1 hour. Control mice were injected with 0.9% NaCl.

### Preparation of serum samples, tissue collection, and plasma total Hcy assay

At the time of sacrifice, blood samples were collected into tubes containing a 1/10 volume of 3.8% sodium citrate, placed on ice immediately. Plasma was isolated by centrifugation at 2500 *g* for 15 min at 4°C. Liver was harvested, snap-frozen and stored at −80°C until use. Plasma total Hcy was assayed by using the fluorimetric high-performance liquid chromatography method described by Fortin and Genest [32]. The inter- and intra-assay coefficients of variation for mean tHcy level were 4.2% and 6.3% respectively and the linearity was from 1 to 100 µM [33].

### Cell Lines and Culture Conditions

Epstein–Barr virus-transformed lymphoblastoid cell lines (LCLs) are derived from healthy individuals and unrelated DS patients, recruited from the Institut Jérôme Lejeune (Paris, France) and the CHU Saint-Etienne (France). Written informed consent was obtained from the participants or from their families, and the French biomedical ethics committee gave its approval for this study (Comité de Protection des Personnes dans la Recherche Biomédicale number 2003–036 and 2005–06). DS was confirmed by karyotyping before and after Epstein–Barr virus transformation. Culture media consisted of Opti-MEM with GlutaMax (Invitrogen, Cergy, France) supplemented with 5% fetal bovine serum from a unique batch and 1% penicillin and streptomycin mix (10,000 U/mL). Cell lines were grown at 37°C in humidified incubators, in an atmosphere of 5% CO_2_. Cells were harvested by centrifugation, washed in 5 mL PBS, followed by another centrifugation, and stored at −80°C.

### Determination of hepatic SAM and SAH concentrations

Levels of SAM and SAH in liver tissues were determined by stable isotope dilution liquid chromatography tandem mass spectrometry in an adapted method from Gellekink et al. [34]. In brief, frozen tissues were homogenized with ice-cold perchloric acid then centrifugated. Supernatant cleanup was performed with solid phase extraction (SPE) columns after neutralization of the acidified samples. The type of SPE cartridges used is phenyl boronic acid (SPE Bond Elut – PBA, 100 mg) from Varian (Courtaboeuf, les Ulis, France). Two isotopically labeled internal standards were used : ^13^C_5_-SAH and D_3_-SAM. Analytes were detected using the transitions m/z 399–250 (SAM), 402–250 (D_3_-SAM), 385–136 (SAH), 390–136 (^13^C_5_-SAH) for quantification and the transitions m/z 399–136 (SAM), 402–136 (D_3_-SAM), 385–134 (SAH), 390–134 (^13^C_5_-SAH) for qualification. The detection limits (signal-to-noise ratio = 3), estimated from the lower calibration point, were 0.4 nmol/L for SAM and 0.6 nmol/L for SAH. The variability of the assay expressed as CVs (n = 4) were 13.3% for SAM and 9.6% for SAH with a plasma pool containing 130 nmol/L of SAM and 11 nmol/L of SAH.

### Western blot analysis

Proteins preparations were subjected to SDS electrophoresis on 7.5% acrylamide gels under reducing conditions and transferred to Hybond-C Extra membrane (GE Healthcare Europe GmbH, Saclay, France). After transfer, membranes were blocked in 10% nonfat dry milk in Tris-saline buffer (1.5 mM Tris, 5 mM NaCl, 0.1% Tween 20) and probed overnight at 4°C with DYRK1A antibody (1/500) (Abnova corporation, Tebu, France). Horseradish peroxidase-conjugated secondary antibody and Western Blotting Luminol Reagent (Santa Cruz Biotechnology, Tebu, France) were used to detect specific proteins. β-actin (1/4000) (Sigma-Aldrich, France) was used as an internal control. Digitized images of the immunoblots obtained using a LAS-3000 imaging system (Fuji Photo Film Co., Ltd.) were used for densitometric measurements with an image analyzer (UnScan It software, Silk Scientific Inc.).

### Enzyme Activity assays

Determination of CBS activity was assayed on 400 µg of total proteins obtained from liver samples, determined by Bradford method, as described [35]. Proteins were incubated for 1h at 37°C with 1 mM of propargylglycine, 0.2 mM of pyridoxal phosphate, 10 mM of L-serine, 10 mM of DL-Hcy, 0.4 mM of SAM, using DTNB (5,5′-dithiobis-(2-nitrobenzoic acid)) based-assay. All the chemical products were obtained from Sigma (Sigma-Aldrich, France). Determination of SAH hydrolase activity was assayed on 300 µg of total proteins obtained from liver samples or lymphoblastoid cell lines following the protocol described by Villanueva and Halsted [36]. Determination of NQO1 activity was assayed on 150 µg of total proteins obtained from liver samples or lymphoblastoid cell lines following the protocol described by Ernster [37], modified by Benson et al. [38].

### RNA extraction and determination of mRNA levels

Total RNA was prepared from mice liver with the Nucleospin® RNA II kit (Macherey-Nagel, Hoerdt). The quantity and purity of the RNA was assessed by measuring absorbance at 260 and 280 nm. Reverse transcription was carried out on 2 µg total RNA as described by the manufacturer (Ambion, UK). The mRNA levels were assessed by quantitative RT-PCR (Q-PCR). cDNA (0.4 µL) was diluted with PCR mix (Light Cycler FastStart DNA Master SYBR Green I Kit, Roche Diagnostics) containing a final concentration of 3 mM MgCl_2_ and 0.5 µM of primers in a final volume of 10 µL. The primers were designed by Primer 3 software. The primers pairs were selected to yield a single amplicon based on dissociation curves. The peptidylprolyl isomerase B (PPIB) mRNA, the hypoxanthine phosphoribosyltransferase (HPRT) mRNA, the fasciculation and elongation zeta protein 1 (fez), and the Zinc finger protein (AB000468) mRNA were used as an endogenous control. Primer sequences were given in the table 5. Q-PCR was performed on total RNA isolated from liver of individual mice or from individual LCLs in a Lightcycler system (Roche Diagnostics). The thermal cycler parameters were as follows : hold for 8 min at 95°C for one cycle followed by amplification of cDNA for 40 cycles with melting for 5 s at 95°C, annealing for 5 s at 65°C and extension for 10 s at 72°C. Each reaction was performed in triplicate. Subsequent assay efficiency calculations were carried out in Light Cycler Relative Quantification Software (Roche Diagnostics). As the efficiency of the target gene and the control genes were comparable, ΔCp analysis of the results allows to assess the ratio of the target mRNA versus control mRNA [39].

**Table 5:** Primer sequences for real-time quantitative reverse transcription-polymerase chain reaction (Q-PCR) (all primers are listed 5′ to 3′).

mRNA	Forward	Reverse
hDYRK1A	TTGAAACGCCACTTTATGTTTCG	CCCAGTAGCACCTCTGGAGACC
hHPRT	GCTGTGGATGCTGTGAAGAA	GCCTCTTCAAGCCAGTAACG
hAB000468	CAAGAAAGCGTCGTGGTGGA	ATCGTCACTGCTCACCACAC
hNQO1	GCCGCAGACCTTGTGATATTCC	GCCACTCTGAATTGGCCAGAGA
hSAHH	TCCGAGGCATCTCTGAGGAGAC	AGCCTGCTACCACCGCTACCTT
mBHMT	GCGTGAGCCAGACGCCTTCATACCTTAG	CCTTTCTGGGGCCAACTCCTCTGCAATC
mDYRK1A	ATCCGACGCACCAGCATC	AATTGTAGACCCTTGGCCTGGT
mfez	TCACGGAGCCAGCATGAATCAG	GGGGAAAAGTCTTGCCGTTCCA
mHPRT	CTCATGGACTGATTATGGACAGGAC	GCAGGTCAGCAAAGAACTTATAGCC
mMAT1a	CAGGTGTCCTATGCCATTGGT	GTAGCACGCAGTCTTCTGGTAGAT
mMS	CCGAGGGATGGAAGCCATTCGAGAAGCA	GTGGCCAACAGCCTTCTTCATGACACGG
mMTHFR	ATGGACTCTGGTGACAAGTGG	AGTGGTCACCTACAGGGTCTCC
mNQO1	GCCATGAAGGAGGCTGCTGT	ATCTGGGCTCAGGCGTCCTT
mPPIB	GGATTTGGCTACAAAAACAGCAA	AGCCAGGCCCGTAGTGCTTC
mSAHH	ACCCTGTTGGGGTTCACTTCCT	TGACATTTGCTCTTGGGAACGA

### Data analysis

Statistical analysis was done with one-way ANOVA followed by Student's unpaired *t*-test using Statview software. In both cases, Student-Newman-Keuls tests were used for multiple pairwise comparaisons. The results are expressed as mean ± SEM. Correlations between Hcy level, DYRK1A protein expression and SAHH activity were determined by using Spearman's rank correlation as data were not normally distributed according to Shapiro – Wilk test. The multivariate analysis was performed according to the principal component analysis (PCA). Data were analyzed using R software (http://www.R-project.org) and considered significant when *p*<0.05. A *p* value of 0.06–0.10 was considered to indicate a strong statistical tendency due to the small sample size.

## 
